# Reviewed article: Perotto JJM, Fonseca MJM, Braga JU. Spatiotemporal analysis of tuberculosis incidence on the border between Brazil and Argentina: a time series study, 2009-2021. Epidemiol Serv Saude. 2025:34;e20240650

**DOI:** 10.1590/S2237-96222025v34e20240650.b

**Published:** 2025-10-20

**Authors:** Gabriel Pavinati

**Affiliations:** 1 Universidade Estadual de Maringá, Maringá, PR, Brazil

## Peer review B

## Questionnaire

Does the manuscript contain new and significant information to justify publication? Yes

Does the Abstract (Summary) clearly and accurately describe the content of the article? No

Is the problem significant and concisely stated? No

Are the methods described comprehensively? No

Are the interpretations and conclusions justified by the results? No

Is adequate reference made to other work in the field? No 

## Manuscript Structure

Length of article is: Adequate

Number of tables is: Adequate

Number of figures is: Adequate

Please state any conflict(s) of interest that you have in relation to the review of this paper. None

Interest: Average

Quality: Good

Originality: Good

Overall: Good

Recommendation: Major Revision

## Comments

Abaixo faço alguns apontamentos que acredito serem úteis para qualificação do manuscrito:

Na introdução, os autores utilizam como aporte teórico o Report TB de 2021. Sugiro substituir pelo que temos de mais recente - Report TB de 2024 (https://www.who.int/teams/global-tuberculosis-programme/tb-reports/global-tuberculosis-report-2024). Também recomendo substituir o Boletim de tuberculose de 2022 pelo de 2024 (https://www.gov.br/aids/pt-br/central-de-conteudo/boletins-epidemiologicos/2024/boletim-epidemiologico-tuberculose-2024/view). Importante acrescer mais algum parágrafo que aponte dados epidemiológicos relativos à incidência da tuberculose nas regiões de fronteiras, especialmente entre Brasil e Argentina. Isso auxiliaria a compreendermos a distribuição do agravo e a justificativa do estudo. Sugestões de estudos que podem ser úteis para essa solicitação (https://doi.org/10.11606/s1518-8787.2024058005531; https://doi.org/10.1590/S2237-96222023000200010; https://pubmed.ncbi.nlm.nih.gov/34076096/). Por fim, recomendo trazer as pacutações/metas nacionais e internacionais em relação à eliminação da tuberculose (The End TB Strategy, Objetivos do Desenvolvimento Sustentável e outros).

Em geral, os estudos apresentados à revista apresentam um objetivo geral. Os pesquisadores optaram por expor três: “caracterizar o perfil demográfico dos pacientes, analisar a evolução temporal e a distribuição espacial da incidência de tuberculose nessa região de fronteira como uma área única e verificar a variação no registro de casos de tuberculose nos períodos pré-pandêmico e pandêmico da COVID-19, no período de 2009 a 2021”. Sugiro a seguinte redação: “analisar os casos de tuberculose na região de fronteira entre Brasil e Argentina, entre 2009 e 2022”. Dessa forma, os autores mantêm a ideia geral do trabalho de maneira mais assertiva e menos redundante. Apesar de não ter enfoque nos anos de pandemia, isso entraria como um achado secundário da pesquisa.

Substituir o descritor “distribuição temporal” por “estudos de séries temporais”. Acredito ser mais apropriado ao que os autores realizaram.

No método, logo no primeiro parágrafo, destacar qual é a unidade de análise do estudo - regiões de saúde, municípios? A Figura 1 não é muito intuitiva para localizar o cenário do estudo. Os municípios considerados são os 48 nominados na Figura C - a imagem está pouco legível na versão em PDF.

Para o cálculo de incidência da tuberculose, utilizou-se duas conceituações distintas - um para cada país? Caso sim, como os autores analisam a influência disso sobre os resultados. É uma limitação do estudo, pensando que no Brasil temos um grande quantitativo de recidivas, especialmente em populações vulneráveis (i.e., imigrantes) - para os casos nacionais isso foi desconsiderando, para a Argentina foi incluído no cálculo das taxas.

Qual foi a fonte de dados para o denominador populacional?

Como os autores trabalharam os casos nacionais que se referiam a imigrantes - foram incluídos como parte da estatística nacional ou desconsiderados da análise?

No excerto: “A distribuição espacial das taxas ajustadas por idade da incidência de tuberculose nos dois períodos do estudo não apresentaram um padrão típico, revelando um padrão de distribuição espacial bastante heterogêneo da doença”. Sugiro que a escrita seja mais direta “a geocodificação das taxas ajustadas por idade da incidência da tuberculose nos dois períodos apresentaram uma distribuição heterogênea”. Seguir a mesma lógica para outras frases semelhantes.

Sugiro que os autores apresentem uma figura ou tabela com a série histórica dos casos de tuberculose. Como as análises de séries temporais não foram significativas para nenhum segmento, é recomendado que os autores se refiram a elas como “estacionárias”, uma vez que não houve padrão significativo. Não se pode, por exemplo, inferir que “primeiro segmento foi de tendência crescente, para Santa Catarina-Brasil entre 2009 e 2016 (VPA=9,3; IC95% −36,8;107,8)”, quando a VPA cruza o valor nulo; nesse caso, a apresentação de uma série histórica traria um aporte descritivo e detalhado sobre as taxas de incidência ano a ano, podendo subsidiar a discussão e fornecendo mais elementos aos leitores para interpretação dos dados.

Da mesma forma, na discussão, no parágrafo inicial de síntese dos achados, os autores destacam: “presença de aglomerados espaciais e tendência de crescimento de casos na área de estudo”. Contudo, novamente, as análises não apresentaram significância, portanto, não se trata de uma tendência crescente, mas sim estacionária. Aqui, reforço a ideia de que uma série histórica poderia fortalecer o estudo, apontando elevação/declínio das taxas de incidência, ano a ano. Notei que no resumo essa afirmação também está presente. Rever.

